# Metabolomic Profiling of Lipids and Fatty Acids: 3 Years Postoperative Laparoscopic Sleeve Gastrectomy

**DOI:** 10.3390/biology10040298

**Published:** 2021-04-05

**Authors:** Charu Sharma, Carine Platat, Salah Gariballa, Shamma Jauaan Al Muhairi, Anas Al Aidaros, Guido Hein Huib Mannaerts, Hamouda Salim Al Afari, Javed Yasin, Osama Y. Al-Dirbashi, Juma Alkaabi

**Affiliations:** 1 Department of Internal Medicine, College of Medicine and Health Sciences, United Arab Emirates University, Al Ain 17666, United Arab Emirates; charusharma@uaeu.ac.ae (C.S.); s.gariballa@uaeu.ac.ae (S.G.); javed.yasin@uaeu.ac.ae (J.Y.); 2 Department of Nutrition and Health, College of Medicine and Health Sciences, United Arab Emirates University, Al Ain 17666, United Arab Emirates; platatcarine@uaeu.ac.ae; 3 Ambulatory Medicine-Medical Affairs, Tawam Hospital, Al Ain 17666, United Arab Emirates; shmuhairi@seha.ae; 4 Department of Pediatrics, College of Medicine and Health Sciences, United Arab Emirates University, Al Ain 17666, United Arab Emirates; a.aidaros@uaeu.ac.ae (A.A.A.); aldirbashi@yahoo.com (O.Y.A.-D.); 5 Surgical Department, Tawam Hospital, Al Ain 17666, United Arab Emirates; GMannaerts@seha.ae; 6 General Surgery Division, Tawam Hospital, Al Ain 17666, United Arab Emirates; hafari@seha.ae; 7 National Reference Laboratory, ICAD 1, Musaffa, Abu Dhabi 92323, United Arab Emirates; 8 Department of Pediatrics, University of Ottawa, 451 Smyth Rd, Ottawa, ON K1H 8M5, Canada

**Keywords:** obesity, laparoscopic sleeve gastrectomy, lipids, fatty acids

## Abstract

**Simple Summary:**

Laparoscopic Sleeve Gastrectomy (LSG) is one of the most effective type of bariatric surgery for weight loss that enables reduction in the size of stomach and associated comorbidities arises from obesity. LSG is well-accepted and safe surgical procedure, but it may lead to malabsorption of nutrients. However, long-term impact of LSG on other risk factors, including blood lipid and fatty acid (FA) profiles, largely remains unknown. The present study aimed to investigate the lipid and plasma FA profiles in patients who underwent LSG in reference to control group with obesity. We determine the markers of inflammation and metabolic risk in these groups by measuring different saturated, monounsaturated, polyunsaturated, and medium-chain FAs. We also estimated the activity of enzymes involved in synthesis of endogenous FA using the product precursor ratios of individual FAs as markers of global metabolic risk. Our study findings showed that patient’s 3-yrs post LSG developed a favorable lipid profile than control with similar metabolic profile in both groups, as determined by the blood FA profile and FA ratios. Our findings showed that LSG offers extended positive effect on the lipid profile in obese population. Though, lifestyle interventions following strict regular follow-up to maintain general health and well-being.

**Abstract:**

Visceral obesity is common in the United Arab Emirates and worldwide. Although laparoscopic sleeve gastrectomy (LSG) leads to effective and sustainable weight loss, its long-term beneficial impact on other risk factors, including blood lipid and fatty acid (FA) profiles, remains unknown. These two profiles were assessed in patients 3 years after undergoing LSG and in LSG candidates (controls). Lipid profiles were measured using the Cobas e411 modular analyzer, and 35 FAs were identified. The age and body mass index were 36.55 ± 8.65 years and 31.49 ± 6.43 kg/m^2^ in the LSG group and 35.44 ± 9.51 years and 32.29 ± 5.38 kg/m^2^ in the control group, respectively. The overall lipid profile was more favorable in the LSG group than in the control group. Total saturated, monounsaturated, and polyunsaturated FAs were similar between the groups, but total medium-chain FAs were more abundant in the LSG group. In endogenous FA synthesis, the estimated activity of C16Δ9 desaturase and Δ5 desaturase decreased, whereas that of elongase increased in the LSG group compared with that in the control group. The benefits of LSG on blood lipid and FA profiles in patients with 3-year LSG may be limited. Hence, lifestyle interventions combined with a long-term and strict regular follow-up regime may be warranted for patients undergoing LSG.

## 1. Introduction

Obesity is a substantial public health concern worldwide, mainly attributed to sedentary lifestyles and inappropriate dietary habits. The United Arab Emirates is a country with one of the largest proportion of obese individuals, with 9.9% of women and 25.6% of men having obesity. Obesity is also associated with comorbidities such as type 2 diabetes, nonalcoholic fatty liver disease, cardiovascular diseases, obstructive sleep apnea, infertility, and some types of cancer [1,2].

Currently employed lifestyle-based strategies have been ineffective in managing obesity [3]. Furthermore, pharmacological treatments are limited and frequently associated with side effects. Therefore, alternative methods that could lead to significant and sustainable weight loss in patients and reduce obesity-related health complications should be identified.

Bariatric surgery, which aims to reduce food consumption by physically restricting the gastric capabilities of the body, has been associated with such effects. One of the most common procedures currently available to patients is laparoscopic sleeve gastrectomy (LSG). LSG is an irreversible restrictive procedure wherein a large portion of the stomach is removed without rerouting the gastrointestinal tract, thereby reducing the total stomach dimensions to approximately 25% from its usual size [4]. Apart from weight reduction, LSG can induce dietary and metabolic changes that can improve obesity-related comorbidities, including diabetes and hypertension, and ultimately, reduce patient’s cardiometabolic risk [5]. Consistently, the total cholesterol (TC) and triglyceride levels generally decrease, and the high-density lipoprotein (HDL) level increases up to 2 years after surgery [6,7]. LSG also likely alters other blood nutritional markers, such as fatty acid (FA) levels. The circulating FA profile is associated with obesity and its related comorbidities. One year after LSG, the levels of saturated FAs (SFA) were shown to decrease, whereas those of n-3 and n-6 polyunsaturated FAs (PUFAs) were shown to increase [8]. n-3 PUFAs and n-6 PUFAs yield antagonistic effects on obesity, supporting an overall benefit of a greater n-3 PUFA/n-6 PUFA ratio [9,10]. n-3 PUFAs and n-6 PUFAs inhibit not only adipocyte growth and fat storage but also inflammation, which is associated with cardiometabolic diseases, including obesity. Inflammation results from cytokine secretion stimulated by eicosanoids. Eicosanoids are lipid mediators derived from n-3 PUFAs and n-6 PUFAs, especially eicosapentaenoic acid (EPA) and arachidonic acid (AA), respectively. In contrast to eicosanoids derived from AA, eicosanoids derived from EPA are associated with the production of anti-inflammatory cytokines. A greater EPA/AA ratio has been related to a lower metabolic risk [9]. Patients with an effective and sustainable weight loss option experience weight regain of up to 15% of their original weight within 2–10 years postoperatively [4]. Noticeably, there is a lack of evidence regarding the beneficial impacts of LSG on lipid and FA profiles, activity of enzymes involved in endogenous FA synthesis, and the global cardiometabolic risk, as suggested by available studies focusing on LSG [7,8]. In addition, none of these studies was conducted over a period of 2 postoperative years. Therefore, it remains unknown if positive LSG exerts a positive effect on lipid and FA profiles beyond 2 postoperative years.

This study aimed to describe and compare the lipid and plasma FA profiles between patients who underwent LSG and a control group comprising patients with obesity to determine if LSG is related to these profiles 3 years postoperatively and how this relationship could be translated in terms of inflammatory status and metabolic risk. The total amount of SFAs, monounsaturated fatty acids (MUFAs), PUFAs, n-3 PUFAs, n-6 PUFAs, and medium-chain FAs (MCFAs) was determined in both the groups. We also estimated the activity of enzymes involved in endogenous FA synthesis by using the product precursor ratios of individual FAs. The n-3 PUFA/n-6 PUFA ratio and the EPA/AA ratio were determined as markers of the global metabolic risk [11].

## 2. Materials and Methods

### 2.1. Participants

This study enrolled patients from the Obesity Center at Tawam Hospital, United Arab Emirates, between December 2008 and June 2013. Patients who underwent LSG at least 3 years ago (n = 71) were included in the study. The controls enrolled in the study were prospective candidates for bariatric surgery (n = 63) with matched age and body mass index (BMI) values. Participants were asked to fast for 10–12 h without any meal specifications or restrictions prior to fasting. A nurse called them 1 day before the blood tests to remind them to fast for 10–12 h and answer any questions from them. The details of the study population are already mentioned elsewhere [12].

### 2.2. Inclusion/Exclusion Criteria

Adult patients who underwent LSG at the Obesity Center at Tawam Hospital at least 3 years ago were eligible. Based on the International Federation for the Surgery of Obesity and Metabolic Disorders guidelines, the criteria for bariatric surgery were as follows: BMI > 30 kg/m^2^ with uncontrolled diabetes, BMI > 35 kg/m^2^ with at least one obesity-related comorbidity, and BMI > 40 kg/m^2^ with or without comorbidities. Comorbidities included type 2 diabetes, nonalcoholic steatohepatitis/hepatic steatosis (fatty liver disease), lymphedema, and hypercholesterolemia (or high triglyceride levels) in patients with other comorbidities, such as sleep apnea requiring continuous positive airway pressure, immediate family history of heart disease and Center for Disease Control and Prevention risk >20%, hypertension requiring three antihypertensive drugs, asthma attack in the previous year or admission or requiring significant corticosteroids or more than two drugs, or brittle asthma or an asthma impeding exercise [13].

In the control group, we included individuals with obesity who were candidates for LSG but had similar age and BMI as the LSG group. The exclusion criteria for the control group were as follows: abuse history; psychiatric, endocrine, gastric, kidney, or liver disease; gut malabsorption syndrome; or other obesity-induced disorders.

All the reported data were collected concurrently for both patients with LSG and the controls, except for sociodemographic characteristics. Sociodemographic data (occupation, marital status, education level, and nationality) were collected at the first visit for all participants, but this time point corresponded to 3 years after surgery for patients with LSG and to the actual first visit for the controls.

The Al Ain Medical District Human Research Ethics Committee approved this study (263/13; #16/008 2016-472). All participants who voluntarily participated provided informed consent. They were invited to visit the hospital at different occasions after surgery for follow-up.

### 2.3. Anthropometry

The participants’ height and weight were measured using a stadiometer and a scale, respectively. In addition, systolic blood pressure (SBP) and diastolic blood pressure were recorded using suitable devices. BMI was calculated as follows: weight in kg/(height in m^2^).

### 2.4. Blood Lipid Profile

Blood was collected in EDTA tubes and centrifuged at 2000 rpm and 4 °C for 20 min, and plasma was collected in Eppendorf tubes and stored at −80 °C. TC, low-density lipoprotein cholesterol (LDL-C), high-density lipoprotein cholesterol (HDL-C), and triglycerides were analyzed using Cobas e411 modular analyzer (Roche Diagnostics, Indianapolis, IN, USA).

### 2.5. Determination of Plasma Fatty Acid Profile

FAs were extracted from plasma using the method described by Dirbashi et al. (2020) [14]. Briefly, 10 μL of patient’s plasma, quality controls, or calibrators were transferred into a borosilicate test tube with a black screw cap (100 × 13 mm^2^, Marienfeld, Germany) and mixed with 60 μL of 5 M HCl and 400 μL of the working internal standard. We incubated the capped tubes at 100 °C for 1 h. Under acidic hydrolysis conditions, free FAs were released from the lipids. The internal standard used in the analysis consisted of the following: (A) 13C4 C8:0 at 7.5 μmol/L, (B) d3-C10 at 37.5 μmol/L, (C) d3-C12 at 75 μmol/L, (D) d3-C14 at 75 μmol/L, (E) d4-C16 at 600 μmol/L, (F) d3-C18 at 300 μmol/L, (G) d3-PRA at 7.5 μmol/L, (H) d3-PHA at 7.5 μmol/L, (I) d4-C22 at 37.5 μmol/L, (J) d4-C24 at 37.5 μmol/L, and (K) d4-C26 at 1.5 μmol/L. The use of appropriate internal standards with stable isotopes resulted in excellent recovery of MCFA C8:0 (98.7%), C10:0 (101%), and C12:0 (98.5%). Analytical imprecision for these FAs expressed as CV% ranged between 0.9% and 6.6%. The samples were further processed using 1 mL of n-hexane, and the supernatants were transferred to another fresh tube. The supernatants were evaporated under nitrogen for derivatization. The residual material was reconstituted in 200 μL of a mixture (2:1:1 *v*/*v*/*v*) of 4-[2-(*N*,*N*-dimethylamino)ethylaminosulfonyl]-7-(2-aminoethylamino)-2,1,3-benzoxadiazole (DAABD-AE; 2 mM in acetonitrile), endocrine-disrupting chemicals (25 mM in water), and 4-dimethylaminopyridine (25 mM in acetonitrile) in order, followed by vigorous shaking for 15 s. Then, we incubated the mixture at 60 °C for 60 min for derivatization and stopped the reaction using the mobile phase. FAs with DAABD-AE reagent and a 0.1-μL aliquot of the resultant mixture were directly analyzed by liquid chromatography with tandem mass spectrometry (LC-MS/MS) using a Shimadzu Nexera X2 chromatography system equipped with a binary pump, thermostated autosampler held at 4 °C, and coupled to LC-MS 8060 triple quadrupole mass spectrometer (Shimadzu, Kyoto, Japan) through electrospray ionization in positive mode.

ESI-MS/MS analysis was performed using N_2_ as a nebulizing (3.0 L/min) and drying gas (10.0 L/min); for collision-induced dissociation, we used argon. Desolvation and ion source temperatures were set at 250 °C and 400 °C, respectively. The capillary voltage was +4.0 kV. Chromatographic separation was achieved on a 2.1 × 50 mm^2^, 1.7 μm C18 column maintained at 40 °C (Acquity UPLC BEH, Waters, Milford, CT, USA) using 10% acetonitrile in water containing 0.5 g/L perfluorooctanoic acid (PFOA) (mobile phase A) and acetonitrile containing 0.5 g/L PFOA (mobile phase B). The gradient program involved varying the proportion of solvent B as follows: 0–1 min, 40%; 1–3 min, 40–65%; 3–3.8 min, 65%; 3.8–6 min, 65–88%; 6–8.5 min, 88%; and 8.5–11 min, 95%. Using 40% mobile phase B, we re-equilibrated the column for 4 min at 0.35 mL/min. Table 1 specifies the analytical parameters employed in this study. Ultimately, we assessed 35 FAs, which included SFAs, MUFAs, PUFAs, n-3 PUFAs, n-6 PUFAs, and MCFAs. In addition, the ratios of n-3 PUFA/n-6 PUFA and EPA/AA were calculated and considered as markers of cardiovascular disease risk [11].

The product precursor ratios of individual FAs in human tissues such as plasma lipids can be used to estimate the activity of enzymes involved in endogenous FA synthesis [15]. Thus, to estimate the activity of C18Δ9 desaturase, C16Δ9 desaturase, Δ6 desaturase, Δ5 desaturase, and elongase, we calculated the following ratios: oleic/stearic, palmitoleic/palmitic, dihomo-γ-linolenic acid/linoleic, AA/dihomo-γlinolenic, and stearic/palmitic FA, respectively.

### 2.6. Statistical Analysis

Data were analyzed using the statistical software package SPSS Statistics version 25 (IBM Corp., Armonk, NY, USA). Means ± SD and percentages were calculated as appropriate. Comparisons between the LSG and control group were performed using the independent *t*-test or chi-square test. The potential effect of sociodemographic data, type 2 diabetes, family history of type 2 diabetes, and blood lipid profile on the relationship between FA profile and LSG was examined using a linear model. *p* < 0.05 indicated statistical significance.

## 3. Results

Table 1 summarizes the general characteristics of the LSG (n = 71) and control (n = 63) groups. The two groups were similar in terms of age; the average age was 36.55 ± 8.65 years in the LSG group and 35.44 ± 9.51 years in the control group. In addition, 97.2% and 100% of the patients who underwent LSG and control individuals were Emirati, 67.6% and 68.3% were married, and the average BMI was 31.49 ± 6.43 and 32.29 ± 5.38 kg/m^2^, respectively. The control group had more women (94.0%), comprising more housewives than employed women, than the LSG group (80.6%). Moreover, the control group comprised more individuals who completed primary/secondary school level of education than the LSG group.

SBP was significantly higher in the control group than in the LSG group. Meanwhile, the incidence of type 2 diabetes was higher in the LSG group than in the control group (9.96% vs. 1.6%), but the family history of type 2 diabetes demonstrated no difference between the two groups. The patients of either group did not take lipid lowering drug. Moreover, the control group participants were not on any medication. However, in the patient groups the medication details are as: B12, eight (8); neurobion, two (2); vitamin D, twenty-one (21); multivitamins, five (5); a combination of calcium and vitamin D, five (5); proton pump inhibitors, seven (7); iron, nine (9); metformin, one (1) and zinc, one (1). None of the patients were taking aspirin or thiamine supplements.

Except TC, LDL-C, HDL-C, and triglyceride levels differed between the groups, with lower triglyceride (*p* < 10^−2^), higher HDL-C (*p* < 10^−3^), and higher LDL-C (*p* < 0.05) levels in the LSG group than in the control group (Table 2). According to the 2018 American Heart Association (AHA) guidelines [16], the average levels of triglycerides and TC were within the desirable range in the two groups but not the average levels of LDL-C and HDL-C.

In the plasma FA profile, 35 FAs were identified. On average, the total amounts of SFAs and MUFAs were similar in the two groups. However, within each category, some FAs differed between the two groups. All SFAs, namely, octanoic acid, decanoic acid, lauric acid, phytanic acid, hexacosanoic acid, and tetracosanoic acid, were more abundant in the LSG group than in the control group. Among MUFAs, the levels of laureloic acid and hexadecenoic acid were lower in the LSG group, whereas the levels of gondoic acid and hexacosenoic acid were higher in the LSG group. The total amount of PUFAs was the same in the two groups. However, the levels of n-3 PUFAs, especially the essential FAs α-linolenic acid, EPA, and docosahexaenoic acid, were lower in the LSG group. Among n-6 PUFAs, the levels of only tetradecadienoic acid and AA differed between the two groups, with lower levels in the LSG group. In both groups, the n-3 PUFA/n-6 PUFA ratio was not balanced but was greater in the control group than in the LSG group. The EPA/AA ratio was the same in the two groups and was in the lower limit of the 0.1–0.2 range recommended by the World Health Organization [17]. Table 3 shows the mean ± SD levels of plasma FAs in the two groups.

MCFAs contain molecules with three–six carbons that are rapidly hydrolyzed in the intestinal tract and transported by portal bloodstream to the liver, where they are readily metabolized. Interestingly, the LSG group had a higher amount of MCFAs than the control group. None of the abovementioned comparisons for FAs was affected by the sociodemographic parameters, presence of type 2 diabetes, family history of type 2 diabetes or lipid profile (data not shown).

Furthermore, the estimated activity of enzymes involved in endogenous FA synthesis demonstrated some differences. Indeed, the estimated activities of both C16Δ9 desaturase, which catalyzes the production of palmitoleic acid by desaturating palmitic acid, and Δ5 desaturase, which catalyzes the production of AA by desaturating dihomo-γ-linolenic acid, were reduced in the LSG group (Table 4).

The estimated activity of elongase was increased in the LSG group, whereas that of both C18Δ9 desaturase and Δ6 desaturase was similar in the two groups.

## 4. Discussion

In this study, we evaluated and compared lipid and plasma FA profiles between adult patients who underwent LSG at least 3 years ago and age- and BMI-matched adult individuals with obesity. The LSG group had lower triglyceride and higher HDL-C levels than the control group. Plasma FA profiles differed for specific FAs, except for SFAs, MUFAs, and PUFAs. Only MCFAs were more abundant in the LSG group than in the control group. Furthermore, the levels of activities of some enzymes involved in endogenous FA synthesis differed between the groups. The n-3 PUFA/n-6 PUFA ratio was lower in the LSG group, whereas the EPA/AA ratio was similar in the two groups, suggesting that the patients who underwent LSG had a higher metabolic risk than the control individuals.

Both n-3 and n-6 PUFAs, including EPA and AA, respectively, are critical for cell membrane function, brain and nervous system development, and eicosanoid production. Eicosanoids derived from n-6 PUFAs are associated with proinflammatory cytokine production, whereas eicosanoids derived from n-3 PUFAs are associated with anti-inflammatory cytokine production. Therefore, an increased amount of n-3 PUFAs, such as EPA, and a decreased amount of n-6 PUFAs, such as AA, indicates an anti-inflammatory status and a decreased risk of developing cardiometabolic diseases, considering that inflammation is a common mechanism involved in the development of these disorders. However, in our study, it was not clear whether a lower n-3PUFA/n-6 PUFA ratio would indicate a higher risk for developing these disorders, as in the case of the LSG group in comparison with the control group. In addition, our results cannot clearly conclude the improvement of the inflammatory status 3 years after undergoing LSG. Indeed, while the EPA/AA ratio was the same in both groups, the n-3 PUFA/n-6 PUFA ratio was lower in the LSG group than in the control group. This result may be related to the fact that the FA profile is the result of various processes, such as dietary intake, intestinal absorption, metabolism, storage, exchanges among compartments, and endogenous synthesis [19]. Endogenous FA synthesis involves various desaturase and elongase enzymes, which could be the principal modulating agents of FA synthesis. The Δ9 desaturase, Δ6 desaturase, and Δ5 desaturase enzymes present a double bond at specific positions (9th, 6th, and 5th carbons from carboxyl terminal) of long-chain FAs. Δ9 desaturase catalyzes the synthesis of MUFAs from SFAs, whereas Δ6 desaturase and Δ5 desaturase catalyzes the synthesis of long-chain n-6 PUFAs and n-3 PUFAs. Elongase is involved in many aspects of endogenous FA synthesis; it adds two carbon units at the carboxyl terminal of FAs. These processes are critical because they generate long-chain and unsaturated FAs, which play major roles in the human body, especially the inflammatory process. The activity of desaturase and elongase is reportedly regulated to maintain an appropriate composition in long and unsaturated FAs [20]. A tight regulation results from the simultaneous stimulation of desaturase expression by transcription factors such as the sterol regulatory element-binding protein, and inhibition by PUFA, desaturase products, and several elongase types, with some preferred SFAs and MUFAs as substrates and others being selective for PUFAs [21,22]. The activities of desaturase and elongase can be estimated by the product precursor ratios of individual FAs in serum lipids. One study reported that the increased estimates of elongase and Δ5 desaturase activities are beneficial 1 year after LSG [23]. However, our study found that the activities of C16Δ9 desaturase and Δ5 desaturase decreased in patients 3 years after undergoing LSG compared with that in the controls because of the lower quantities of palmitoleic acid and AA. As supported by some evidence, the association between the increased activity of Δ9 desaturase and Δ6 desaturase and decreased activity for Δ5 desaturase and elongase activity leads to various metabolic risks and inflammatory status [15]. Recently, high Δ6 desaturase activity was found to be related to a metabolically healthy status [18]. Finally, the LSG group had lower AA levels, indicating a healthier metabolic profile than the control group, but their differences in desaturase and elongase activities would rather indicate a degraded metabolic status and a proinflammatory condition. This contradictory conclusion is also supported by the similarity of the EPA/AA ratio between the two groups. High values of this ratio are related to a decreased risk of developing many diseases, including cardiovascular disease [24]. Thus, 3 years after undergoing LSG, patients would be in a transitory phase, as characterized by a progressive degradation of the levels of beneficial and anti-inflammatory FAs and a negative change of the enzymatic activity during endogenous FA synthesis.

Compared with other surgical procedures, especially Roux-en-Y gastric bypass, LSG is more efficient in changing the triglyceride and HDL-C levels but not the TC and LDL-C levels [25,26], as supported by our results. Further, the lipid profile was apparently more beneficial among patients 3 years after undergoing LSG than among adults with obesity with matched age and BMI. Considering that patients in previous studies had lower triglyceride levels and higher HDL-C levels up to 2 years postoperatively [6,7], LSG could have an even longer positive impact on some of the lipid profile parameters, especially HDL-C, up to 3 years after the procedure. This effect might be explained by the improvement of HDL-C efflux capacity [27]. This effect on HDL-C is crucial, given that HDL-C particles are suspected to have an effect beyond their role as a carrier and could mediate diverse mechanisms promoting cardiovascular health [28].

**Figure 1:**
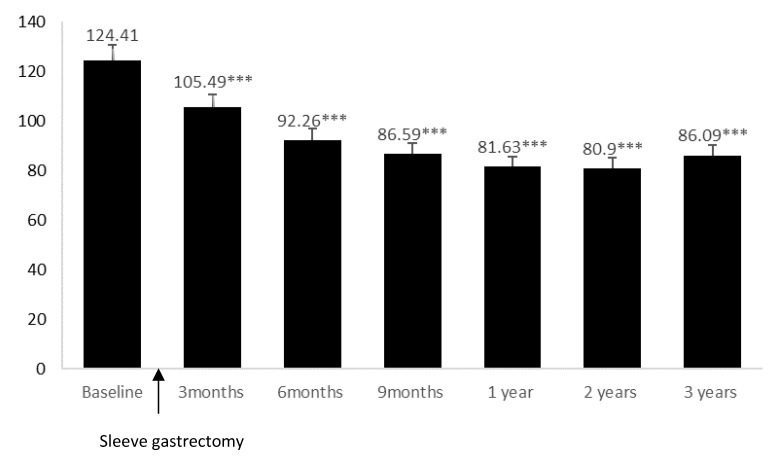
Weight change in patients before (baseline) and up to 3 years after undergoing laparoscopic sleeve gastrectomy, presented as means ± SD *. Comparisons were performed with baseline using the independent *t*-test. *** *p* < 10^−3^.

However, the HDL-C levels 3 years after LSG were not within the recommended range by the AHA. HDL-C improvement induced by bariatric surgery is closely related to weight loss extension [29]. Interestingly, the LSG group appeared to regain weight, starting from 3 years postoperatively (Figure 1). Therefore, LSG could help in maintaining HDL-C at a higher level despite weight gain. This result supports the existence of a maximum HDL-C increase at 2 years after LSG, as previously suggested by Genua et al. (2020) [26].

One major result related to the plasma FA profile is the higher MCFA levels among the LSG patients. Similarly, the level of some MCFAs increased in a previous study, but the increment occurred 6 months after Roux-en-Y gastric bypass [30]. Although MCFAs are mainly derived from milk and other dairy products, a greater dietary intake of MCFAs does not explain the increased serum level despite that food intake restriction and nutrient absorption reduction only take place a few months after the surgery. MCFAs can be synthesized in the liver [31] and prevent fat deposition by enhancing fat oxidation and thermogenesis [32]. Therefore, the weight loss induced by bariatric surgery could be related to an upregulation of the hepatic production of MCFAs. However, 3 years after LSG, MCFA intake could increase despite not receiving any nutritional support. In parallel, weight loss vanishes, and weight regain seems to start (Figure 1). MCFAs also promote lipogenesis, and this function could explain weight regain emergence [31]. Therefore, the modulation of the lipid balance by MCFAs and the impact on weight could depend on the endogenous or dietary origin of MCFAs, and 3 years after LSG, the positive effect of LSG on weight loss through the increase of endogenous MCFA synthesis could be exceeded by the lipogenic effect of dietary MCFA. However, this notion remains unconfirmed.

The levels of αlinolenic, EPA, and docosahexaenoic acids were lower in the LSG patients than in the controls. Generally, n-3 PUFAs are less abundant in patients with obesity than in individuals without obesity, probably attributed to the oxidative stress related to obesity. In this study, the difference between the LSG patients and the controls with obesity could be explained by a reduced dietary intake of these FAs because of the gastric resection; another possible explanation was the potential long-chain FA uptake by adipocytes as it has already been emphasized more than a year after LSG [33]. In parallel, two n-6 PUFAs, namely, tetradecadienoic acid and AA, were reduced in the LSG patients. The n-3 and n-6 PUFAs exert antagonistic effects on inflammation and are differently related to the risk of developing diseases, such as cancer, highlighting the importance of having a high n-3/n-6 PUFA ratio [34]. Consequently, the decrement of these two n-6 PUFAs is rather a positive effect. However, overall, the metabolic risk, as evaluated by the EPA/AA ratio, remained similar between the two groups.

The dietary data, relevant markers of the metabolic risk, and inflammatory biomarkers should be assessed to support the relationships identified between the LSG, blood lipid profile, and FA profile and to confirm the proposed related mechanisms. Despite the potential influence on the comparison between the FA profile, education level, and occupation status, which were all different between the LSG patients and the controls, an indirect effect through dietary habit modifications cannot be totally excluded. Nevertheless, it provides new insights into the impact of LSG on lipid and plasma FA profiles in comparison with individuals with obesity. Indeed, our results were obtained at a quite long time, that is, 3 years, after the surgical procedure compared with the results of most of the studies conducted within the first year following the surgery [7,8,23]. Moreover, our analysis of 35 different FAs provides a unique and extensive view of the plasma FA profile among the LSG patients and individuals matched by BMI. Further, our study is limited by one-time blood sampling. Identifying the changes in FA profile at different time periods would provide a more thorough information. Nonetheless, the present study provided valuable information because we could confirm many of the serum FA profile changes that are positively related to 3 years after surgery.

## 5. Conclusions

Patients with 3-year LSG gained a more favorable lipid profile than individuals with obesity before LSG, but both groups had a similar metabolic profile, as estimated by the blood FA profile and FA ratios. Thus, LSG provided a prolonged positive effect on the lipid profile. However, our study highlighted that 3 years after LSG is a critical period of time for managing the metabolic profile of patients who underwent LSG. 

## Figures and Tables

**Table 1:** General characteristics of patients 3 years after undergoing laparoscopic sleeve gastrectomy (LSG) and control individuals, presented as mean ± SD or %.

	LSG Group	Control Group
	(n = 71)	(n = 63)
Age (years)	36.55 ± 8.65	35.44 ± 9.51
Sex (%)		
Female	80.6	94.0
Occupation (%)		
Employed	67.6	17.5 ***
Housewife	15.5	66.7
Unemployed/Retired	16.9	15.9
Nationality (%)		
GCC countries	2.8	0
UAE	97.2	100.0
Marital status (%)		
Married	67.6	68.3
Divorced/Single/Widowed	32.4	31.8
Level of education (%)		
Primary/Secondary school	45.1	540 *
University/Graduated	53.5	36.5
Illiterate	1.4	9.5
Height (cm)	162.08 ± 12.54	159.33 ± 6.90
Weight (kg)	86.09 ± 21.71	81.97 ± 14.52
BMI (kg/m^2^)	31.49 ± 6.43	32.29 ± 5.38
SBP (mmHg)	113.76 ± 12.62	118.92 ± 12.82 *
DBP (mmHg)	69.93 ± 10.84	73.54 ± 8.01
Type 2 diabetes (%)	9.9	1.6 **
Family history of diabetes (%)	76.1	66.7

Abbreviations: GCC: Gulf cooperation council, BMI = body mass index, SBP = systolic blood pressure, DBP = diastolic blood pressure. Comparisons were performed using the chi-square or independent t-test, as appropriate. * *p* < 0.05, ** *p* < 10^−2^, *** *p* < 10^−3^.

**Table 2:** Lipid profile of patients 3 years after undergoing laparoscopic sleeve gastrectomy (LSG) and of control individuals, presented as mean ± SD.

	LSG Group	Control Group
	(n = 71)	(n = 61)
TC (mmol/L)	4.91 ± 0.93	4.67 ± 0.71
LDL-C (mmol/L)	3.05 ± 0.87	2.78 ± 0.67 *
HDL-C (mmol/L)	1.53 ± 0.33	1.02 ± 0.30 ***
TG (mmol/L)	0.79 ± 0.36	1.03 ± 0.55 **

Abbreviations: TC = total cholesterol, LDL-C = low-density-lipoprotein cholesterol, HDL-C = high-density-lipoprotein cholesterol, TG = triglycerides. Comparisons were performed using ANOVA. * *p* < 0.05, ** *p* < 10^−2^, *** *p* < 10^−3^.

**Table 3:** Plasma fatty acid (µmol/L) profile in patients 3 years after undergoing laparoscopic sleeve gastrectomy (LSG) and in control individuals, presented as means ± SD.

	LSG Group	Control Group
	(n = 71)	(n = 63)
Octanoic acid C8:0	79.11 ± 6.62	68.29 ± 6.40 ***
Decenoic acid C10:1	3.44 ± 1.47	3.22 ± 1.45
Decanoic acid C10:0	19.93 ± 1.86	18.77 ± 1.78 ***
Laureloic acid C12:1	2.04 ± 0.57	2.24 ± 0.52 *
Lauric C12:0	60.04 ± 15.08	52.53 ± 22.19 *
Myristoleic acid C14:1	6.38 ± 4.35	7.17 ± 4.22
Myristic acid C14:0	85.65 ± 33.65	82.67 ± 39.70
Tetradecadienoic acid C14:2 n-6	23.58 ± 10.42	28.13 ± 10.07 *
Palmitoleic acid C16:1 n-7	138.61 ± 77.04	181.01 ± 91.38 **
Palmitic acid C16:0	1862.92 ± 429.32	1926.67 ± 514.09
Stearedonic acid C18:4 n-3	1.65 ± 1.46	1.69 ± 1.53
αLinolenic acid C18:3 n-3	9.42 ± 4.37	12.52 ± 7.53 **
γLinolenic acid C18:3 n-6	16.92 ± 12.12	19.32 ± 10.36
Linoleic acid C18:2 n-6	1826.11 ± 412.33	1702.98 ± 460.91
Oleic acid C18:1 n-9	1157.44 ± 357.78	1097.16 ± 299.79
Stearic acid C18:0	586.03 ± 129.40	573.71 ± 130.04
Pristanic acid C19:0	0.64 ± 0.24	0.58 ± 0.17
Phytanic acid C20:0	1.99 ± 0.79	1.43 ± 0.53 ***
EPAC20:5 n-3	30.53 ± 17.39	42.82 ± 26.83 **
AA C20:4 n-6	290.17 ± 115.80	371.21 ± 118.16 ***
dihomo-γ-Linolenic Acid C20:3 n-6	40.19 ± 13.87	40.52 ± 13.68
Eicosadienoic acid C20:2 n-6	5.82 ± 1.79	5.46 ± 1.73
Gondoic acid C20:1	5.61 ± 1.70	4.65 ± 1.23 ***
Arachidic acid C20:0	16.99 ± 4.06	16.69 ± 3.63
Docosahexaenoic acid C22:6 n-3	79.28 ± 40.69	117.11 ± 49.85 ***
Docosapentaenoic acid C22:5 n-3	13.29 ± 6.58	13.28 ± 4.29
Docosatetraenoic acid C22:4 n-6	22.93 ± 9.44	22.35 ± 7.28
Docosatrienoic acid C22:3 n-3	0.33 ± 0.11	0.31 ± 0.10
Docosadienoic acid C22:2 n-6	0.85 ± 0.25	0.80 ± 0.23
Docosenoic acid C22:1	1.39 ± 0.43	1.39 ± 0.42
Docosanoic acid C22:0	44.59 ± 10.59	41.08 ± 10.87
Nervonic acid C24:1	70.37 ± 20.71	65.72 ± 20.49
Tetracosanoic acid C24:0	42.30 ± 8.93	36.37 ± 10.56 **
Hexacosenoic acid C26:1	0.62 ± 0.20	0.51 ± 0.18 **
Hexacosanoic acid C26:0	0.61 ± 0.11	0.56 ± 0.13 *
SFAs	2798.15 ± 590.03	2817.36 ± 686.88
MUFAs	1385.88 ± 437.28	1362.81 ± 381.11
PUFAs	2361.08 ± 540.62	2378.87 ± 605.08
n-6	2226.58 ± 495.79	2191.14 ± 555.42
n-3	134.50 ± 64.04	187.72 ± 78.05 ***
n-3 PUFA/n-6 PUFA	0.06 ± 0.02	0.09 ± 0.03 ***
MCFAs	164.55 ± 19.36	144.79 ± 26.48 ***
EPA/AA	0.10 ± 0.03	0.11 ± 0.06

Abbreviations: SFAs = saturated fatty acids, MUFAs = monounsaturated fatty acids, PUFAs = polyunsaturated fatty acids, MCFAs = medium-chain fatty acids, EPAs = eicosapentaenoic acid, AAs = arachidonic acid. The independent *t*-test was used to perform comparisons. * *p* < 0.05, ** *p* < 10^−2^, *** *p* < 10^−3.^

**Table 4:** Estimated activities of C18Δ9 desaturase, C16Δ9 desaturase, Δ6 desaturase, Δ5 desaturase and elongase in patients 3 years after undergoing laparoscopic sleeve gastrectomy and control individuals, presented as means ± SD.

	LSG Group	Control Group
	(n = 71)	(n = 63)
C18Δ9 desaturase	1.97 ± 0.35	1.92 ± 0.29
C16Δ9 desaturase	0.07 ± 0.03	0.09 ± 0.03 ***
Δ6 desaturase	0.02 ± 0.01	0.02 ± 0.01
Δ5 desaturase	7.46 ± 2.27	9.71 ± 3.43 ***
Elongase	0.32 ± 0.03	0.30 ± 0.04 *

Comparisons were performed using the independent *t*-test. * *p* < 0.05, *** *p* < 10^−3^. Fatty acid ratios used to estimate enzymatic activities were oleic/stearic ratio for C18Δ9 desaturase activity, palmitoleic/palmitic ratio for C16Δ9 desaturase activity, dihomo-γ-linolenic acid/linoleic ratio for Δ6 desaturase activity, arachidonic/dihomo-γlinolenic for Δ5 desaturase activity, and stearic/palmitic ratio for elongase activity [15,18].

## Data Availability

Not applicable.
